# Selenium Disulfide from Sustainable Resources: An Example of “Redneck” Chemistry with a Pinch of Salt

**DOI:** 10.3390/ma17235733

**Published:** 2024-11-23

**Authors:** Eduard Tiganescu, Shahrzad Safinazlou, Ahmad Yaman Abdin, Rainer Lilischkis, Karl-Herbert Schäfer, Claudia Fink-Straube, Muhammad Jawad Nasim, Claus Jacob

**Affiliations:** 1 Division of Bioorganic Chemistry, School of Pharmacy, Saarland University, 66123 Saarbruecken, Germany; s9edtiga@stud.uni-saarland.de (E.T.); shsa00006@stud.uni-saarland.de (S.S.); yaman.abdin@uni-saarland.de (A.Y.A.); 2 Working Group Enteric Nervous Systems (AGENS), University of Applied Sciences Kaiserslautern, Amerikastrasse 1, 66482 Kaiserslautern, Germany; rainer.lilischkis@hs-kl.de (R.L.); karlherbert.schaefer@hs-kl.de (K.-H.S.); 3 INM–Leibniz Institute for New Materials, 66123 Saarbrücken, Germany; claudia.fink-straube@leibniz-inm.de

**Keywords:** hydrogen sulfide, local economy, nanomaterials, selenium disulfide, spring water, sustainable synthesis, waste-to-value strategy

## Abstract

Selenium disulfide (often referred to as SeS_2_) encompasses a family of mixed selenium-sulfide eight-membered rings, traditionally used as an anti-dandruff agent in shampoos. SeS_2_ can be produced by reacting hydrogen sulfide (H_2_S) with selenite (SeO_3_^2−^) under acidic conditions. This chemistry is also possible with natural spring waters that are rich in H_2_S, thus providing an avenue for the more sustainable, green production of high-quality SeS_2_ particles from an abundant natural source. The orange material obtained this way consists of small globules with a diameter in the range of 1.1 to 1.2 µm composed of various Se_x_S_8−x_ chalcogen rings. It shows the usual composition and characteristics of a Se-S interchalcogen compound in EDX and Raman spectroscopy. Since the mineral water from Bad Nenndorf is also rich in salts, the leftover brine has been evaporated to yield a selenium-enriched salt mixture similar to table salt. As the water from Bad Nenndorf—in comparison to other bodies of water around the world—is still rather modest in terms of its H_2_S content, especially when compared with volcanic waters, this approach may be refined further to become economically and ecologically viable, especially as a regional business model for small and medium-sized enterprises.

## 1. Introduction

Selenium and sulfur are two redox-active non-metals at the heart of many powerful natural redox-modulating compounds, ranging from highly reducing natural products such as ovothiols and selenoneine to oxidizing, electrophilic disulfides and disulfide-*S*-oxides (e.g., thiosulfinates, thiosulfonates), such as allicin [1,2,3,4,5]. The selenium–sulfur bond is especially redox-active and often catalytic and has been the focus of many studies on biological redox-cycles, for instance in glutathione peroxidase (GPx) enzymes [6,7,8,9]. Notably, selenium and sulfur can also theoretically “do without” a carbon-skeleton as far as their appearances, stabilities and redox chemistries are concerned. Catenation, their ability to form chains and rings with themselves and other elements of the chalcogen group, ensures that they are not “alone”, and some of these inorganic sulfur–selenium molecules can reach sizes of eight or more atoms.

Selenium disulfide, an orange solid composed of a medley of eight member Se_x_S_8−x_ rings, is a prime example of this type of “chalcogen-only” chemistry, devoid of any additional organic ballast [10]. Due to its average chemical composition of around 1:2 for Se and S, it is often referred to somewhat misleadingly as SeS_2_ and structurally represented as S=Se=S in analogy to SeO_2_ or SO_2_, which in fact is structurally incorrect, since SeS_2_ consists of the eight-membered rings linked via single sulfur–sulfur, sulfur–selenium and selenium–selenium bonds, with sulfur and selenium in the formal oxidation states of zero [11,12].

SeS_2_ can be synthesized in different ways, ranging from the rather alchemistic melting and mixing of elemental selenium and sulfur in a crucible to more controlled reactions, such as in the commercial method for its synthesis, which employs sodium sulfide (Na_2_S) solution acidified with glacial acetic acid and SeO_2_. Although this industrial method already avoids organic solvents, it still relies on commercial Na_2_S, which itself is produced by the reduction of sodium sulfate (Na_2_SO_4_) either at the expense of carbon or hydrogen [13,14,15]. From a green chemistry standpoint, it is therefore tempting to explore alternative, more sustainable methods, which may substitute industrial Na_2_S with naturally occurring H_2_S.

Indeed, H_2_S is quite abundant and common in nature, and a staggering 100–324 million tons of this gas are released from natural sources each year, including inorganic volcanic fumes and waters, as well as organic H_2_S produced in oceans, swamps, bogs, cesspits and honey-wagons [16,17,18,19]. In the Pacific Ring of Fire, volcanoes such as the Kawah Ijen in the East Java region of Indonesia are famous for producing H_2_S at a concentration of 15.7 mM in fumarolic discharge in the air [20]. The “Shah Field” in the United Arab Emirates holds approximately 480 billion cubic meters of sour gas reserves with around 23% H_2_S content [21]. Sulfate-reducing bacteria represent another important source for organic H_2_S production, especially in anoxic waters [22]. Table 1 provides a list of such sulfur-rich natural springs, which may be considered “accessible” natural sources of H_2_S.

Although Germany possesses no active volcanoes, there are areas of extinct volcanic activity and mineral springs rich in salts and H_2_S [28,29]. Among them, the “Neue Landgrafenquelle” in the spa town of Bad Nenndorf in the Lower Saxony region of Germany stands out due to its relatively high content of total sulfur of around 140 mg L^−1^ and a H_2_S concentration of 2.4 mM and indeed, “Bad” is referring to the German word for ‘spa’, or ‘bath’, which does not necessarily imply any pungent sulfury odor.

Guided by the goal of valorizing this natural H_2_S-rich water as a “green” supply of a chemical substance in a waste-to-value and zero-waste process, we have explored its practical uses in the synthesis of SeS_2_ on one hand and Se salt as by-product on the other hand.

## 2. Materials and Methods

### 2.1. Collection and Characterization of H_2_S-Rich Spring Water from Bad Nenndorf

The sulfur-rich water was collected on the early morning of the 6 December 2023 at approximately 8:15 a.m. from the mineral spring in a field near Bad Nenndorf in the Niedersachsen (Lower Saxony) region of Germany. A total of 50 L of this water was stored in ten canisters, each with a capacity of 5 L. The weather at the time of collection was snowy, with a temperature of 0 °C, relative humidity of 96%, and atmospheric pressure of 1011 hPa. The geographic coordinates of the collection site were 52.379947° N, 9.424150° E, and it was 138.4 m above sea level. The geological composition of the ground was primarily Turonian chalks [28]. The freshly collected water samples presented an average pH of 6.6.

Upon arrival at the laboratory of the University of Saarland, the water samples collected were analyzed for their sulfide contents, elemental compositions, and pH values. The Methylene Blue (MB) assay was performed to quantify the sulfide content according to the protocols described in the literature [30,31]. The mineral composition of the water was determined via Atomization Optical Emission Spectrometry with Inductively Coupled Plasma (ICP-OES) using an Ultima 2 tool (Horiba Jobin-Yvon, Longjumenau, France) coupled with a Czerny–Turner-type monochromator with a focal length of 1 m.

### 2.2. Synthesis of SeS_2_

SeO_2_ (1.33 g, 12 mM) was added to the stirred solution of 10 L of sulfide-rich water (24 mM of H_2_S) previously acidified with concentrated hydrochloric acid (HCl, 37%, 4 mL), which adjusted the pH of the reaction mixture to 4.0. The reaction mixture was stirred for two hours at room temperature to obtain an orange precipitate, which was then collected by vacuum filtration. The precipitate was washed with distilled water (100 mL) to remove any unreacted SeO_2_ and traces of spring water. The product was dried at room temperature and stored in the dark until further use. The filtrate was used further, as described in Section 3.4.

### 2.3. Chemical Composition of SeS_2_

The product was subjected to extensive physico-chemical characterization, including CHNS analysis, Energy-Dispersive X-ray Spectroscopy (EDX), Raman spectroscopy, and ICP-OES. CHNS was performed on a Vario MICRO cube CHN-elemental analyser (Elementar GmbH, Langenselbold, Germany). EDX analysis was carried out using a ZEISS Supra 40 field emitter microscope (Carl Zeiss NTS GmbH, Oberkochen, Germany) attached to a Bruker Quantax EDX system (Bruker Nano GmbH, Berlin, Germany). Raman spectroscopy was performed using a Renishaw InVia microscope (Wotton-under-Edge, Gloucestershire, UK) coupled with an excitation laser adjusted to a 532 nm wavelength. Commercially available SeS_2_ (Merck, Darmstadt, Germany) served as a reference material for comparing the composition of the material.

### 2.4. Zetasizer Measurements for Size and Surface Potential

The material was also analyzed using a Zetasizer Nano ZS (Malvern Instruments, Malvern, UK) to measure size and Zeta potential. The particle sizes were recorded after 1 h and 2 h only, since subsequent readings pointed to the significant sedimentation and aggregation of the particles after 2 h, so the particle size could no longer be recorded by the Zetasizer.

## 3. Results

In summary, SeS_2_ was synthesized successfully from spring water from the well at Bad Nenndorf and SeO_2_ at pH 4.0 under mild reaction conditions. As expected, the resulting orange material consisted of microscopic globular structures composed of various Se_x_S_8−x_ rings. The remaining brine left over during the reaction was converted into selenium-enriched (natural) salt, which could be considered for human consumption. The filtrate comprising water and HCl could be recycled, as anticipated for a zero-waste approach.

### 3.1. Analysis of Sulfur Rich Water

The spring water collected at the mineral well at Bad Nenndorf was analyzed for its relevant mineral composition, and elemental analysis confirmed the presence of higher amounts of sodium (12.2 g L^−1^), sulfur (1.552 g L^−1^), and calcium (1.5 g L^−1^), as shown in Table 2.

**Figure 1:**
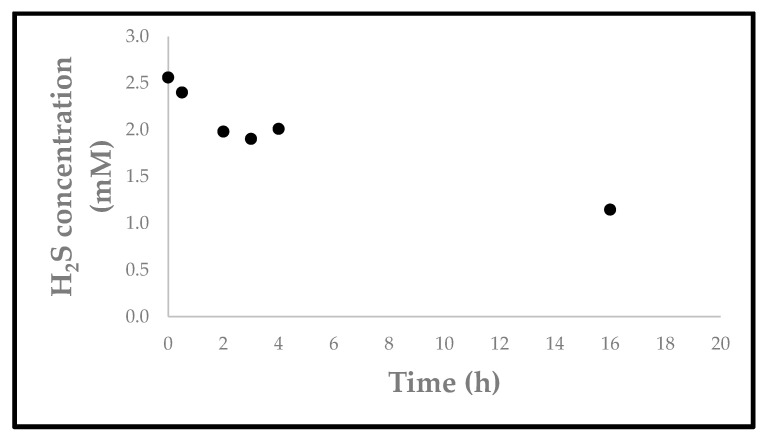
The H_2_S concentration present in the water samples gradually decreases as affirmed using the MB assay.

In line with German regulations on spas and water used for human maceration, a detailed analysis of such natural waters must be performed regularly, and the relevant information from the latest certified analysis carried out by Laborunion Prof. Höll & Co. GmbH, Bad Elster, Germany in 2018 is therefore provided in the Supplemental Information. Unlike this certification analysis, the analysis performed as part of this study focuses on the ingredients relevant for the subsequent chemical reaction, which are in line with the certified values as far as sulfide content (2.4 mM) and pH value (6.6) are concerned. Notably, whereas the pH is stable for days, the sulfide content decreases slowly over time, mostly via the escape of H_2_S, which, for instance, is able to diffuse through such plastic containers (Figure 1).

### 3.2. Synthesis of SeS_2_ and Structural Confirmation

**Figure 2:**
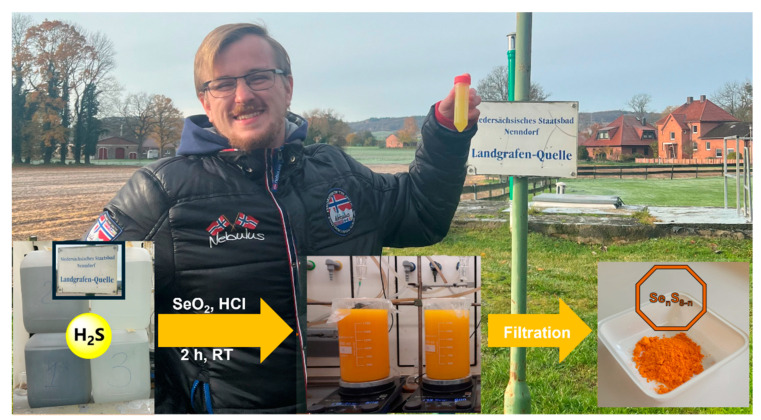
The water was collected from the underground spring in a field near Bad Nenndorf in northern Germany. A preliminary reaction was carried out “redneck style” at the source of origin, and an immediate change in color confirmed the feasibility of the synthesis. The figure also represents the chemistry carried out in the laboratory and shows a photograph of the orange material obtained.

Since the H_2_S content of collected water gradually decreases, as shown in Figure 1, the reaction with SeO_2_ was performed promptly after collection. The reaction occurred solely at lower pH values; thus, HCl was added to the spring water prior to the addition of SeO_2_ to acidify the reaction mixture from the natural pH of 6.6 to pH 4.0. As shown in Figure 2, the reaction mixture turned opaque orange almost immediately after the addition of 0.5 equivalents of SeO_2_, indicating a fast reaction leading to a colloidal product and precipitation. This reaction was highly efficient and could even be performed outside the laboratory in a small Falcon tube “in the field” (Figure 2). It is important to point out that the minerals in the water did not interfere significantly with the reaction. Approximately 2.3 g of the compound was obtained from 20 L of water with a yield of about 66%.

The SeS_2_ precipitate was collected by vacuum filtration, washed with distilled water, and dried at 50 °C in the oven. A combination of CHN-S analysis for sulfur and ICP-OES analysis for selenium content confirmed an elemental selenium-to-sulfur ratio of 1:2 (recalculated as an atomic percentage from the mass percentage of the samples), as shown in Table 3. The analytical properties of the SeS_2_ obtained from spring water therefore did not differ notably from the ones of commercially obtained SeS_2_, including elemental composition and melting point. This is not trivial, as sulfur and selenium can take positions freely in such eight-membered rings and thus, in theory, many different ratios from 1:7 to 7:1 are possible.

**Figure 3:**
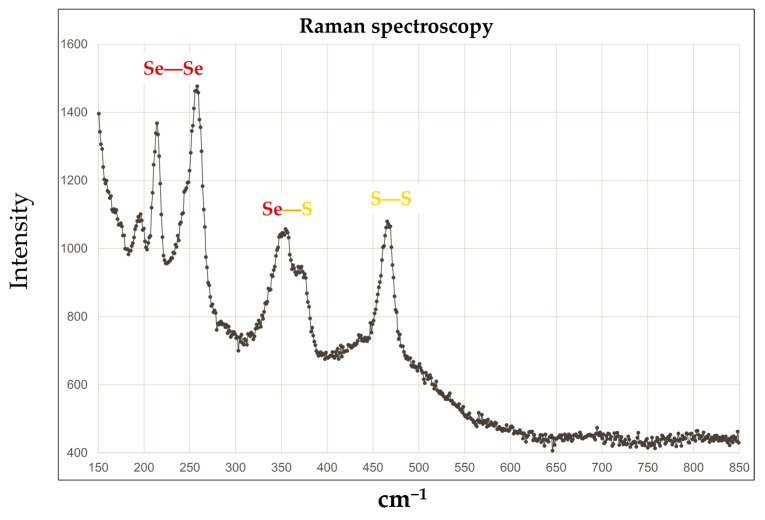
Raman spectroscopy confirmed the presence of S-S, S-Se, and Se-Se bonds.

In order to confirm the presence of a genuine selenium disulfide compound, and not simply that of a physical mixture of elemental selenium and sulfur, Raman spectroscopy was employed, and it confirmed the presence of Se-Se, Se-S, and S-S bonds (Figure 3). Based on the Se-S ratios, which were also found by EDX (Table 3), and under the assumption that the material consists of eight-membered rings, finding a plethora of structures in the “circle of eight” was possible, and indeed likely, especially Se_2_S_6_ and Se_3_S_5_ rings.

### 3.3. Microscopic Properties of the Material

**Figure 4:**
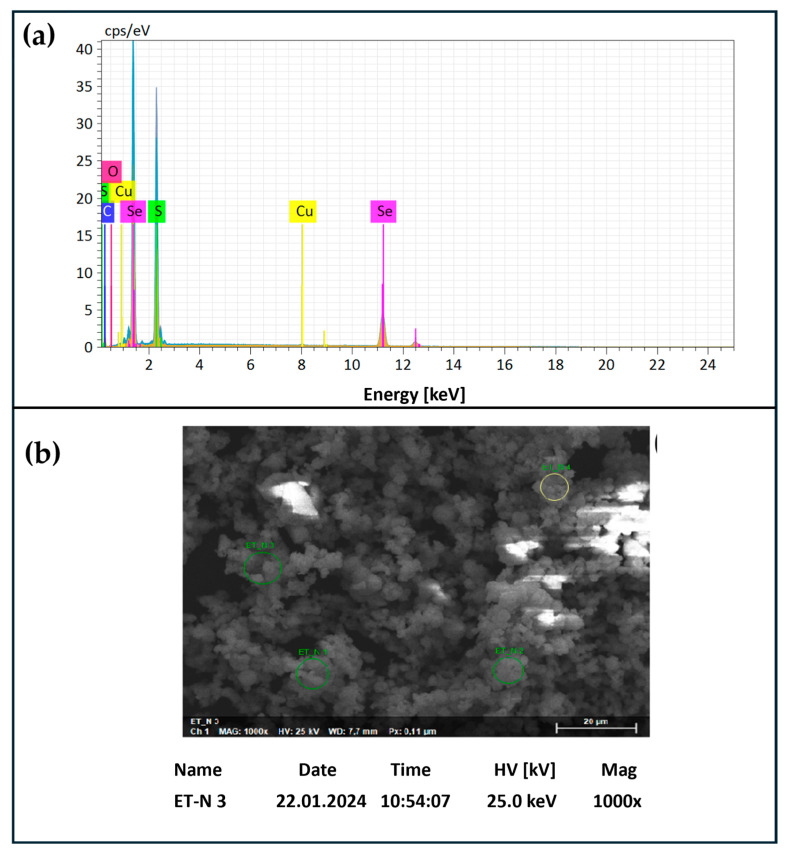
SeS_2_ was analyzed to determine chemical composition using EDX coupled to SEM. EDX confirmed the presence of selenium and sulfur at a ratio of around 1:2 (Panel (**a**)), while the SEM image showed the presence of (aggregated) globular material (Panel (**b**)).

Upon formation, samples of SeS_2_ particles were analyzed by Dynamic Light Scattering (DLS) using a Zetasizer, which confirmed the formation of a precipitate with an average particle size of around 1 µm in diameter (PDI = 0.180) after one hour of reaction, which continued to increase over time. Notably, the Zeta potential, indicative of the surface charge of the particles, was around −4.77 mV, which may also explain why the material tended to aggregate and precipitate from the solution rather readily. To confirm these DLS measurements and to evaluate the elemental composition of the precipitated materials, SEM, in combination with EDX (Figure 4, Panel a), was utilized and indicated the presence of small globular selenium disulfide-rich objects, with sizes in the high nanometer to low micrometer range (Figure 4, Panel b).

### 3.4. Se Salt

**Figure 5:**
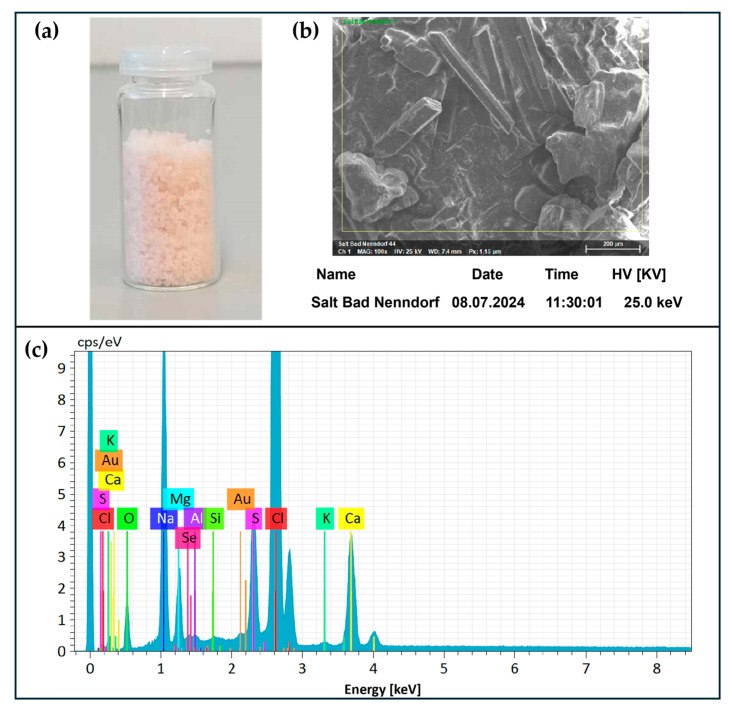
The filtrate was evaporated at 50 °C to obtain salt (Panel (**a**)), which was analyzed by SEM (Panel (**b**)) coupled with EDX (Panel (**c**)) to quantify the elements present in the salt. EDX confirmed the presence of selenium at about 0.40% *w*/*w* (dry weight), as compared to the overall salt composition.

The spring water from Bad Nenndorf is not only rich in H_2_S but also contains high percentages of salt, namely NaCl. The Mediterranean Sea’s surface water, for instance, comprises around 12.3 g L^−1^ of sodium, which is remarkably similar to the water sample from Bad Nenndorf, with a sodium content of 12.2 g L^−1^ (Table 2) [32]. Therefore, the filtrate of the reaction was collected and evaporated in an oven at 50 °C until dryness. A total of 25 mL of this filtrate yielded approximately 3 g (12% *w*/*w*) of salt (Figure 5).

This “leftover” salt from the reaction consisted primarily of sodium (20.35% *w*/*w*) and chloride (44.76% *w*/*w*). Additionally, the resulting salt was rich in calcium (8.78% *w*/*w*) and magnesium (3.81% *w*/*w*) yet low in sulfide (4.52% *w*/*w* for total sulfur). Since the reaction to form SeS_2_ did not go to completion, traces of selenium in the range of 0.40% *w*/*w* (dry weight) were found (Table 4). Apart from this, once dissolved in distilled water, the pH was almost neutral (pH = 7.9 of a 0.9% isotonic salt solution), indicating that the H_2_S initially present (and not reacted with SeO_2_) and the HCl added had both escaped.

## 4. Discussion

To conserve valuable natural resources and to protect the environment, several industries are currently searching for either renewable, recyclable, or valorizable materials. This also applies to inorganic substances, which in many respects are more difficult to (re)produce than organic ones. For instance, in the European Union (EU), phosphate mining is projected to be substituted by chemical recycling initiatives from 2026 onwards [32,33,34,35]. Nitrogen and sulfur may follow suit; thus, it is imperative to incorporate a greater number of sustainable production methods into the chemical industry to deter waste production and deleterious effects on our environment. Sulfur-rich mineral water may, quite literally, run down the drain if not utilized for its abundant elemental content and would often require steeply priced treatment to remove H_2_S, for instance by oxidation with H_2_O_2_ [36,37]. The mineral water from Bad Nenndorf is such a source - . though its H_2_S content is modest in comparison to other international sources, it is sufficient to facilitate the reaction with SeO_2_ in order to produce SeS_2_ to a good quality and yield.

The SeS_2_ produced shows a composition and characteristics close to those of its commercial equivalent, ranging from its elemental composition to vibrations in the Raman spectra characteristic of Se-Se, Se-S, and S-S bonds. Although one cannot rule out the presence of additional elemental selenium and sulfur in such mixtures, the SeS_2_ obtained seems surprisingly pure thanks to its precipitation from the reaction mixture. The compound itself is easy to handle in air and when immersed in water.

SeS_2_ is commonly considered for its action against scalp irritation and has served as a standard active ingredient in anti-dandruff shampoos for several decades, eventually earning some global prominence as the agent that saves the world from alien invasion in the blockbuster film “Evolution” (2001) [38,39,40].

As for the waste-to-value strategy and its underlying “redneck” chemistry, both may still require optimization; yet, they demonstrate that the quality of the mineral water, while rich in many components aside from sulfide, is still “clean” enough to allow the chemical reaction to proceed. Notably, whereas the purification of the water, performed in order to harvest its individual components, may be tedious and costly, the SeS_2_ reaction results in precipitation, which is key for purity, as the product can be removed by simple filtration. Furthermore, the reaction does not yield any side products and the remaining filtrate can be valorized further to form a Se salt.

Interestingly, the NaCl content in this Se salt is comparable to sea salt, which contains around 30.6% sodium and 55.2% chloride [41]. As for taste, the corresponding author took the exceptional step to carefully taste a pinch of this salt for the sake of science. The taste is generally amenable and not much different from that of normal table salt. Aside from human consumption, this salt may be more commonly used for bathing and cosmetic purposes, similar to commercially available Dead Sea salts [42,43].

From an ecological perspective, our proposed procedure serves as an attractive, green alternative, since it does not require or generate harmful chemicals, replaces one component of SeS_2_ production with a natural waste product, and, at the same time, also removes H_2_S from the water. H_2_S removal, for instance via oxidation with H_2_O_2_, is demanding and often required by law, for instance in H_2_S-rich water from abandoned coal mines in Germany and abandoned gas wells in Canada [24,44,45,46]. Although it has not yet been possible to replace commercial SeO_2_ with a natural product, this is a matter that may also be addressed by considering wastes from copper mining and refineries [47,48]. Concerning the HCl employed during SeS_2_ synthesis, it can be recycled during the process, and the water can eventually be recycled during the drying of Se salt.

**Figure 6:**
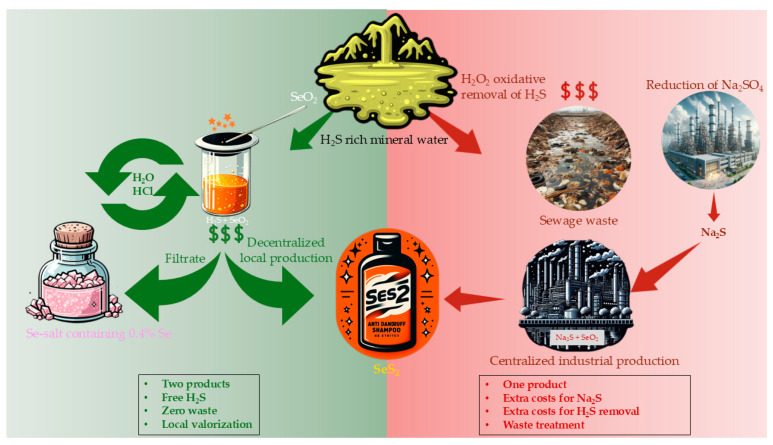
H_2_S springs may serve as sources for the production of value-added products, such as SeS_2,_ and avoid wasting this natural resource as sewage. This strategy not only opens up the door for boosting local economies but also, as a true “hat trick”, decreases the environmental burden posed by the chemical treatment of H_2_S-rich water.

Economically speaking, solely using mineral water from Bad Nenndorf for the large-scale production of SeS_2_ is, of course, not possible. Here, other richer, more concentrated sources need to be considered, such as the Solec-Zdrój “Malina” spring in Poland, where over 400 kg of H_2_S can be harvested annually, considering a theoretical flow rate of 1 L min^−1^. This natural source may be sufficient to replace Na_2_S, which has a cost of several hundred Euros per ton. Then again, small(er)-scale local production in spa towns with similar mineral springs to Bad Nenndorf, such as Aachen or Bad Wiessee in Germany, may still be attractive, as it could empower local communities and contribute to their overall economic development. These local natural spring spas may, for instance, launch their own proprietary cosmetic products, such as shampoos, or produce their own brands of Se salt on a commercial and larger scale; furthermore, these locations could market these products, allowing for sales to cover production costs and incentivize a small profit margin. This may support the local and regional economy around such spa towns, which are often located in rural areas. Eventually, these smaller and somewhat niche SeS_2_ business opportunities for small and medium-sized enterprises might become quite lucrative, considering that mineral water is often discarded as waste and must be purified, most commonly via oxidation at an added cost (Figure 6).

## 5. Conclusions

In summary, our studies have confirmed that it is possible and indeed quite attractive to leverage natural (re)sources in chemical reactions, as illustrated by our method for producing SeS_2_. Our proposed procedure can be seen as yet another example and proof-of-concept for a carefully devised and designed green and sustainable strategy to transmute waste into value, with the sustainable benefits of protecting the environment and serving as a boon to local economies. Aside from utilizing sources of spring water of inorganic origin, our future directions will focus upon procuring H_2_S from organic sources, such as wastewater, sewage, and bacterial cultures, which are able to produce H_2_S either from organic materials during fouling, by reducing sulfite (SO_3_^2−^), or naturally abundant sulfate (SO_4_^2−^) [49,50,51,52]. We also intend to look for opportunities to “freeload” SeO_2_, although this may turn out to be more challenging.

In line with our aims of endorsing sustainability and, at the same time, employing local resources, the strategy of collecting nearby, readily available materials (including, but not limited to, waste bins, sewage treatment plants, soil, and air) and using them as chemical reagents could significantly gain momentum. Yet, this requires a carefully devised “redneck” chemistry equipped to handle initially dirty, impure mixtures of substances, on the one hand, and possible byproducts and “left-overs”, on the other hand. In our example, the precipitation of insoluble SeS_2_ has done the trick, i.e., it has enabled the separation of considerably pure SeS_2_ from the minerals in the water and the water itself. Ultimately, our methods have bolstered support for the further use of the Se-rich salt as part of a zero-waste strategy.

In the future, it will prove encouraging to employ similar local (re)sources and a chemistry that is able to blend natural materials with traditional reactions in order to provide further high-value, low-cost products. Precipitation or the production of gases may circumvent some of the issues associated with turning “dirty” waste into clean products. Eventually, these clean products will be obtained from “impure” (re)sources by a cleverly designed yet robust “redneck” chemistry, possibly on smaller scales, for local production and regional waste-to-value chains alike.

## Figures and Tables

**Table 1:** Natural (re)sources of H_2_S with relevant sulfide concentrations.

Name	Country	H_2_S Content (mM L^−1^)	Literature
Solec-Zdrój	Poland	23.1	[23]
Legacy gas wells in southwestern Ontario	Canada	11.7	[24]
Ust’Kachka Resort Spring, Perm Krai	Russia	7	[25]
Bad Nenndorf	Germany	2.4	[26]
Mamlaha	Iraq	2.3	[27]

**Table 2:** The concentrations of selected inorganic elements found in the mineral water from Bad Nenndorf (see the Supplementary Materials for comparison).

Elements	B	Ca	Mg	Na	Si	Sr	S
**Concentration (mg L^−1^)**	7	1500	420	12,200	5	34	1552

**Table 3:** The elemental composition of the selenium disulfide powder obtained compared to a selenium disulfide sample from a commercial supplier.

Methods	CHN-S	EDX		ICP-OES
Elements	S (%)	S (%)	Se (%)	Se (%)
Synthesized compound	48.25	48.70	51.30	52.90
Reference compound	43.36	43.80	56.20	56.00

**Table 4:** Elemental composition of Se salt, as estimated by employing EDX.

Elements found	O	Na	Mg	Al	Si	S	Cl	K	Ca	Se
**Composition (% *w*/*w*)**	16.24	20.35	3.81	0.26	0.10	4.52	44.76	0.25	8.78	0.40

## Data Availability

The raw data supporting the conclusions of this article will be made available by the authors on request.

## Supplementary Materials

The following supporting information can be downloaded at: https://www.mdpi.com/article/10.3390/ma17235733/s1, Table S1: Physical and physico-chemical analysis of the Landgrafen spring from Bad Nenndorf; Table S2: Elemental composition analysis of the Landgrafen spring from Bad Nenndorf; Table S3: Undissociated and gaseous substances found in the Landgrafen spring from Bad Nenndorf.
