# Supplementary material for: Selenium Disulfide from Sustainable Resources: An Example of “Redneck” Chemistry with a Pinch of Salt
Source: Materials (Basel). 2024 Nov 23;17(23):5733. doi: 10.3390/ma17235733 (PMC11642483; doi:10.3390/ma17235733)
Supplement: Supplementary file 1 [file materials-17-05733-s001.zip › materials-3221841-supplementary.pdf]

Table S1. Physical and physico-chemical analysis of the Landgrafen spring from Bad Nenndorf

Designation of the measured variables	Unit	Measured value
Temperature of the water at the sampling point	°C	21.0
Air temperature (outside)	°C	23.0
PH value at sampling laboratory		6.4<br>6.8
Conductivity: laboratory 25 °C	µS.cm^{-1}	155000
Redox voltage at: withdrawal temperature against standard hydrogen electrode at 20 °C	mV<br>mV	-304<br>-93
Oxygen content	mg.L^{-1}	0.07
Density at 20 °C	g.cm^{-1}	1.0807

Table S2. Elemental composition analysis of the Landgrafen spring from Bad Nenndorf

	**Mass concentration mg.L^{-1}**	**Equivalent concentration mmol.L^{-1}**	**Equivalent share %**
Lithium	1.89	0.272	0.013
Sodium	43200	1879	91.947
Potassium	81.9	2.005	0.102
Magnesium	662	54.47	2.666
Calcium	2120	105.8	5.176
Strontium	43.8	1.000	0.049
Iron	0.14	0.005	0.000
Manganese	< 0.10	-	-
Ammonium	16.9	0.937	0.046
**Total**	**46127**	**2.044**	**100**
Fluoride	0.57	0.030	0.001
Chloride	68800	1941	95.220
Bromide	37.1	0.464	0.023
Iodide	0.82	0.006	0.000
Sulfate	4450	92.65	4.546
Nitrite	0.028	0.001	0.000
Nitrate	3.4	0.055	0.003
Hydrogen carbonate	222	3.638	0.179
Hydrogen sulfide	18.7	0.565	0.03
Hydrogen phosphate	0.05	0.001	0.000
**Total**	**73533**	**2.038**	**100**

Table S3. Undissociated and gaseous substances found in the Landgrafen spring from Bad Nenndorf

Designation of the measuring grids	Unit	Measured value
**Undissociated substances:**		
Meta-Boric acid	$mg.L^{-1}$	16.1
Silica	$mg.L^{-1}$	5.4
Total dissolved solids	$mg.L^{-1}$	119682
**Gaseous substances:**		
Free dissolved carbon dioxide	$mg.L^{-1}$	< 25
at 0° C and 1013 hPa	mL	< 13
Hydrogen sulfide	$mg.L^{-1}$	84.2
**Total sum of listed substances**	**$mg.L^{-1}$**	**119766**
